# Evaluation of secretome biomarkers in glioblastoma cancer stem cells: A bioinformatics analysis

**DOI:** 10.1002/cnr2.2080

**Published:** 2024-07-05

**Authors:** Ehsan Jangholi, Hoda Ahmari Tehran, Afsaneh Ghasemi, Mohammad Hoseinian, Sina Firoozi, Seyed Mohammad Ghodsi, Mona Tamaddon, Ahmad Bereimipour, Mahmoudreza Hadjighassem

**Affiliations:** ^1^ Brain and Spinal Cord Injury Research Center Neuroscience Institute, Tehran University of Medical Sciences Tehran Iran; ^2^ Department of Neurosurgery Shariati Hospital, Tehran University of Medical Sciences Tehran Iran; ^3^ Department of Medical Education Qom University of Medical Sciences Qom Iran; ^4^ Department of Public Health School of Health, Fasa University of Medical Sciences Fasa Iran; ^5^ School of Medicine Kermanshah University of Medical Sciences Kermanshah Iran; ^6^ Chronic Disease Research Center Endocrinology and Metabolism Population Sciences Institute, Tehran University of Medical Sciences Tehran Iran; ^7^ Department of Biological Sciences and BioDiscovery Institute University of North Texas Denton Texas USA

**Keywords:** bioinformatics analysis, biomarker, cancer stem cells, gene expression profiles, glioblastoma

## Abstract

**Background:**

Glioblastoma (GBM) is a malignant brain tumor that frequently occurs alongside other central nervous system (CNS) conditions. The secretome of GBM cells contains a diverse array of proteins released into the extracellular space, influencing the tumor microenvironment. These proteins can serve as potential biomarkers for GBM due to their involvement in key biological processes, exploring the secretome biomarkers in GBM research represents a cutting‐edge strategy with significant potential for advancing diagnostic precision, treatment monitoring, and ultimately improving outcomes for patients with this challenging brain cancer.

**Aim:**

This study was aimed to investigate the roles of secretome biomarkers and their pathwayes in GBM through bioinformatics analysis.

**Methods and Results:**

Using data from the Gene Expression Omnibus and the Cancer Genome Atlas datasets—where both healthy and cancerous samples were analyzed—we used a quantitative analytical framework to identify differentially expressed genes (DEGs) and cell signaling pathways that might be related to GBM. Then, we performed gene ontology studies and hub protein identifications to estimate the roles of these DEGs after finding disease‐gene connection networks and signaling pathways. Using the GEPIA Proportional Hazard Model and the Kaplan–Meier estimator, we widened our analysis to identify the important genes that may play a role in both progression and the survival of patients with GBM. In total, 890 DEGs, including 475 and 415 upregulated and downregulated were identified, respectively. Our results revealed that *SQLE*, *DHCR7*, *delta‐1 phospholipase C* (*PLCD1*), and *MINPP1* genes are highly expressed, and the *Enolase 2* (*ENO2*) and *hexokinase‐1* (*HK1*) genes are low expressions.

**Conclusion:**

Hence, our findings suggest novel mechanisms that affect the occurrence of GBM development, growth, and/or establishment and may also serve as secretory biomarkers for GBM prognosis and possible targets for therapy. So, continued research in this field may uncover new avenues for therapeutic interventions and contribute to the ongoing efforts to combat GBM effectively.

## 1 INTRODUCTION

Glioblastoma (GBM) is the most common and, unfortunately, most malignant brain tumor, which is more common in men.^1^ There is no clear boundary between the tumor and the brain tissue, and the tumor cells penetrate the normal brain tissue over long distances, so complementary therapies are often used to control the growth and spread of this cell.^2^ These complementary therapies include radiation therapy and chemotherapy.^2^ Meanwhile, cancer stem cells (CSCs) play a significant role in resistance to treatment and can even cause the recurrence of high‐intensity disease and secondary tumors.^3^, ^4^ Therefore, finding signal pathways and proteins, especially secretory biomarkers, can be a new key to treating the GBM microenvironment.^4^ Although CSCs make up about 1% of the total tumor cell population, they could lead to the recurrence of the disease and/or the formation of secondary tumors.^5^, ^6^, ^7^

The relationship between secretome biomarkers and GBM is a dynamic field of study within cancer research. GBM cells release a diverse array of proteins into their microenvironment, forming the secretome, which plays a pivotal role in tumor progression. Researchers focus on identifying specific secreted proteins as biomarkers, aiming to harness their diagnostic potential for early detection and accurate characterization of GBM. These biomarkers may offer insights into disease aggressiveness and prognosis, shaping therapeutic strategies. Furthermore, the secretome influences the tumor microenvironment, impacting cellular interactions and immune responses.^8^ As potential therapeutic targets, secretome biomarkers hold promise for developing personalized treatments and monitoring changes in the secretome during therapy may provide valuable information on treatment response and resistance. In essence, investigating the secretome in the context of GBM presents a comprehensive approach to understanding the disease's biology and exploring avenues for improved diagnostic and therapeutic interventions.^9^

Glioblastoma cancer stem cells (GSCs) present a formidable challenge in the treatment of GBM, a highly aggressive and infiltrative brain cancer. These stem‐like cells within the tumor exhibit self‐renewal and differentiation capabilities, contributing to tumor heterogeneity, recurrence, and therapeutic resistance. The resilience of GSCs to conventional treatments, such as radiation and chemotherapy, poses a significant obstacle, as these therapies may target bulk tumor cells while leaving GSCs largely unaffected.^10^ Additionally, the plasticity of GSCs allows them to adapt to changing microenvironments and evade immune responses, further complicating treatment strategies. Developing effective therapies against GSCs necessitates a deep understanding of their molecular mechanisms, signaling pathways, and interactions within the tumor microenvironment. Overcoming the challenges posed by GSCs requires innovative therapeutic approaches that specifically target and eliminate these resilient CSCs, potentially leading to more successful and durable outcomes in the management of GBM.^11^

Over the past decade, bioinformatics knowledge has been instrumental in finding biomarkers from the genome to the proteome in various cancers.^12^, ^13^, ^14^ In this study, using continuous and integrated bioinformatics analyses, we investigated the gene expression profile of GBM CSCs and isolated specific pathways and proteins that are involved in the extracellular matrix (ECM) secretory processes, ECM, and microenvironment of GBM.

## 2 METHODS AND MATERIALS

### 2.1 GBM stem cell gene expression profile datasets

In this study, the GSE146698 database from the Gene Expression Omnibus (GEO) database (https://www.ncbi.nlm.nih.gov/geo) was selected. This dataset contains six samples that included two groups of three members in the form of GBM tissue‐derived stem cells and a control cell. The platform used in this dataset was GPL17077 Agilent‐039494 SurePrint G3 Human GE v2 8 × 60 K Microarray 039381.

### 2.2 Preparation of gene expression profile data for additional analysis

**FIGURE 1:**
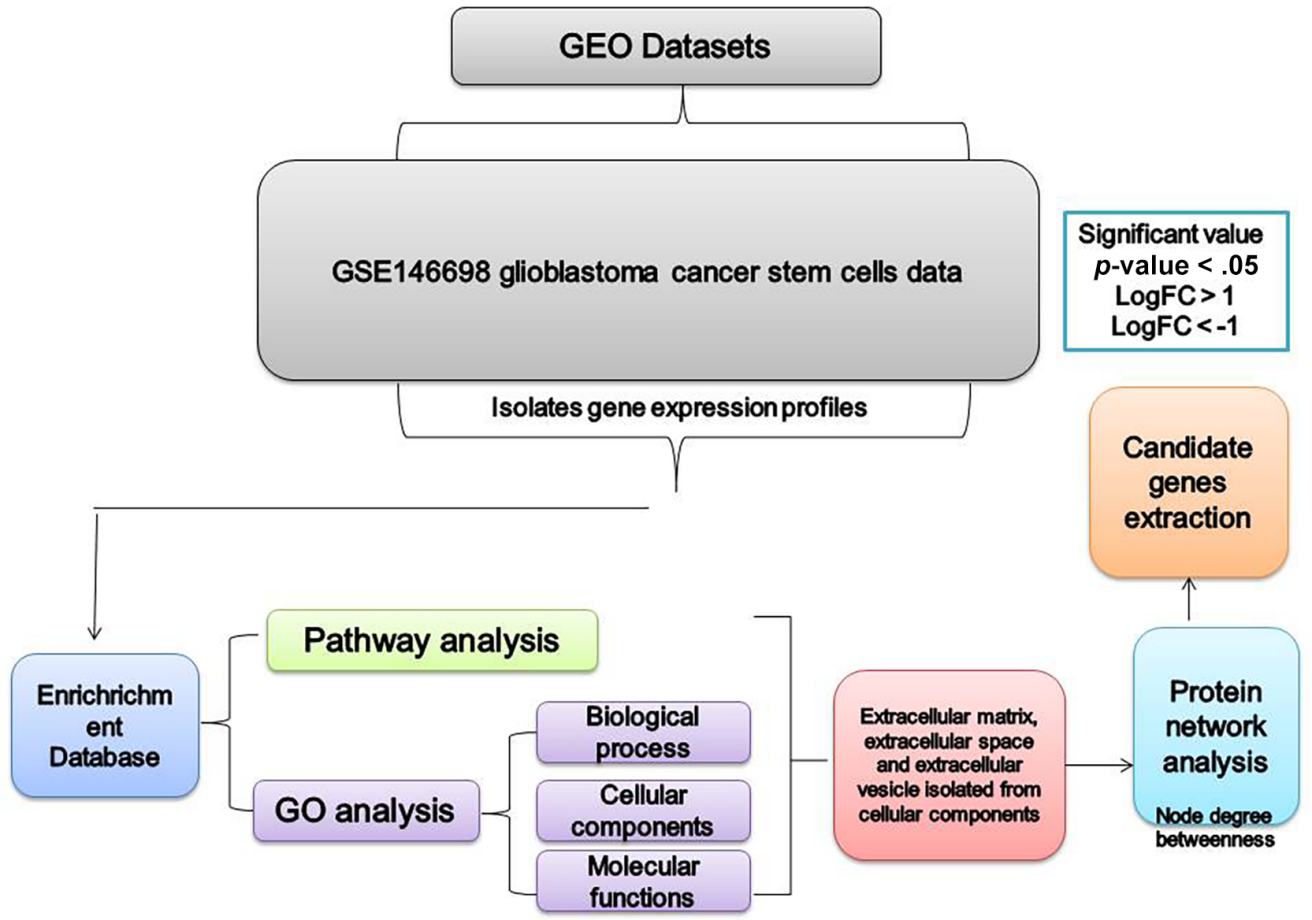
Schematic pathway of how to perform bioinformatics analysis. GEO, Gene Expression OmnibusIA; GO, gene ontology.

The GSE146698 dataset was isolated using GEO2R analysis, then genes with *p* < .05, logFC <−1, and logFC>1 were isolated, and then genes with high‐ and low‐expression were classified. Finally, it was prepared for other analyses (Figure 1).

### 2.3 Determined signaling pathways and the gene ontology

From the GSE146698 dataset, genes subject to differential expression were analyzed separately with the Enrichr database (https://amp.pharm.mssm.edu/Enrichr), and with the help of Kyoto Encyclopedia of Genes and Genomes (KEGG), the related signal pathways and its genes were selected. In addition, by using the Enrichr and PANTHER databases (http://www.pantherdb.org/geneListAnalysis.do), biological processes (BPs), molecular functions (MFs), and cellular components (CCs) were evaluated. In this study, genes with high expression were more important due to selecting appropriate biomarkers, especially in CCs. The next step evaluated 10 genes with the highest and lowest expressions separately. Also, *p* < .05 was considered to study the signal pathway and GOs.

### 2.4 Investigation of the relationship between proteins

Selected genes from the signal and GO pathways were uploaded to the network analyst and section STRINGS database (https://string-db.org), and a network of linkages between proteins was obtained. To better display the data, we used Cytoscape software (version 3.7.1).

### 2.5 Evaluation of gene candidates in clinical data

**FIGURE 2:**
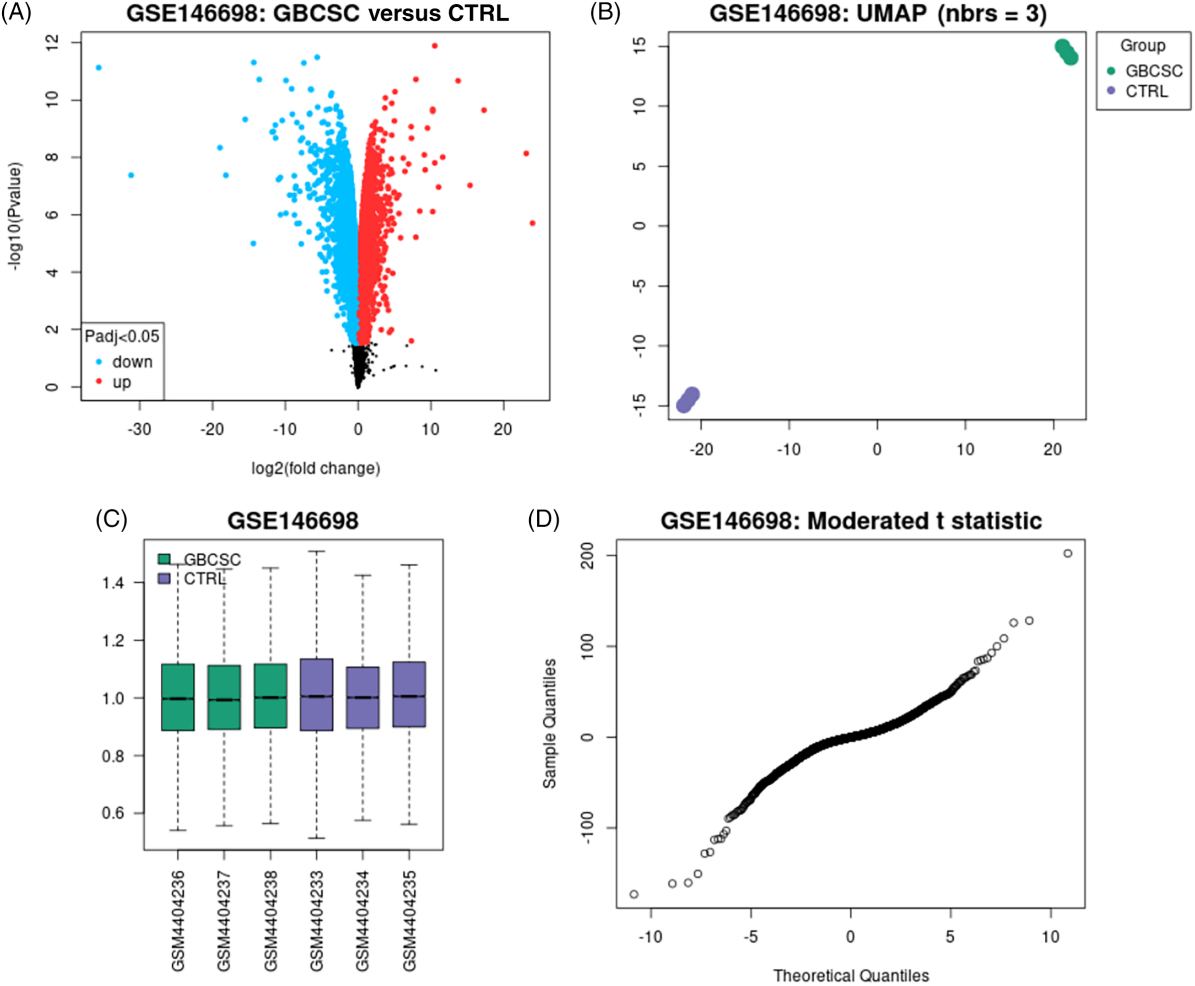
General information on the expression profile of the GSE146698 dataset gene associated with glioblastoma cancer stem cells. Accordingly, the volcano diagram (A) shows the gene expression differentiation between the two groups. Also, the PCA diagram (B) indicates that the density and placement of the samples of each group next to each other are good similarities between the data, which are a good option for analysis. (C) The box diagram of the samples of each group. (D) Degree of differentiation of gene expression in each group. Figure 3: evaluation of signal pathways marked for low‐ (A) and high‐expressions (B) genes.

After selecting the most important communication proteins in this section, we entered them in the GEPIA database and evaluated them with clinical data on gene expression and survival (Figure 2).

### 2.6 Ethical consideration

This study was approved by the Research Ethics Committees of the Neuroscience Institute (approval code: IR.TUMS.NI.REC.1401.023).

## 3 RESULTS

### 3.1 Apoptosis, microenvironments pathways, ECM receptor, and mitogen‐activated protein kinase pathway

**FIGURE 3:**
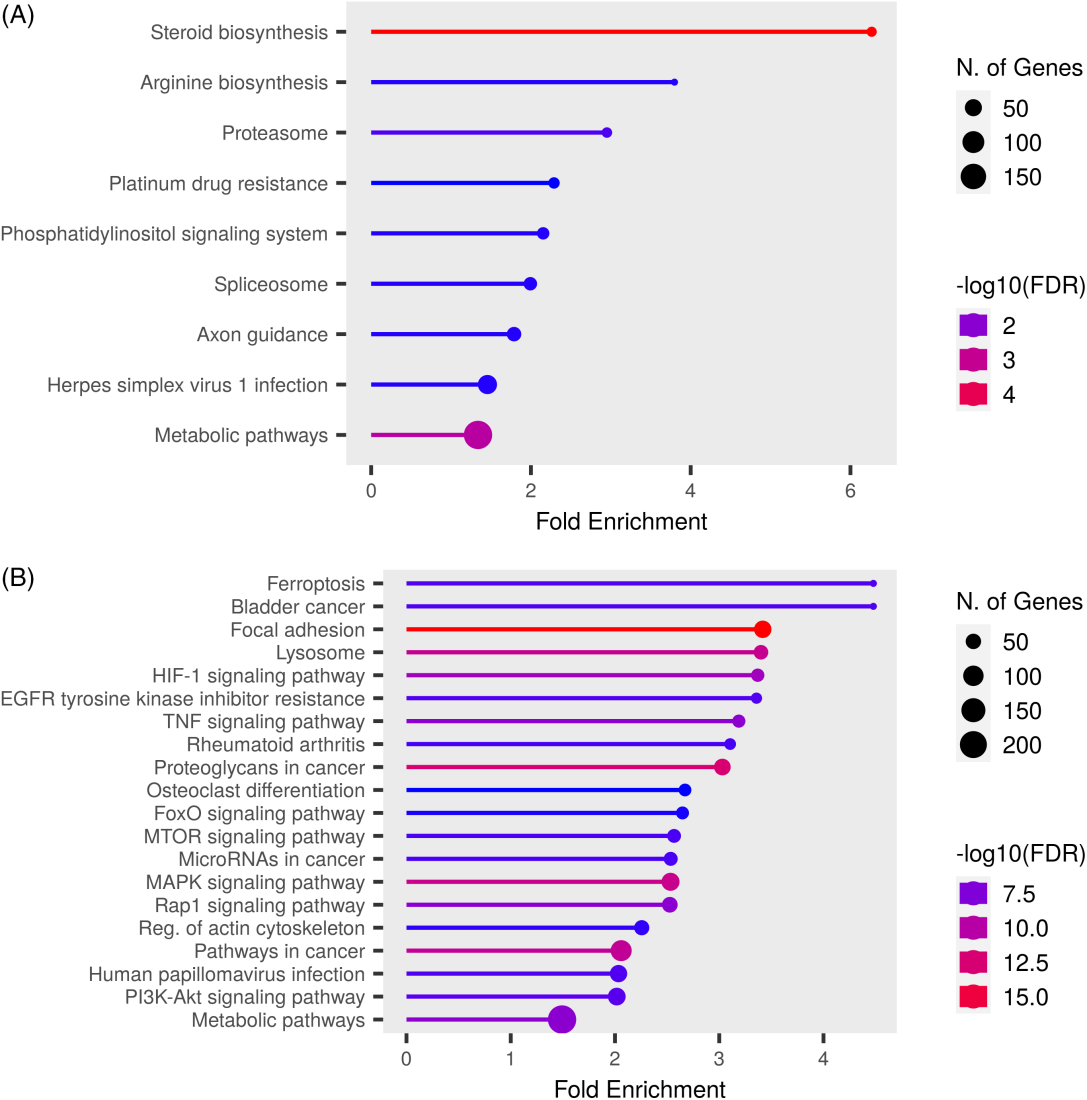
Evaluation of signal pathways marked for low‐ (A) and high‐expressions (B) genes.

Analysis of the GSE146698 gene expression profile showed that 1250 high‐expression and 1030 low‐expression genes could play a role in the critical pathways of GBM progression. Apoptosis, insulin receptor, P53 signaling, microenvironments signaling, ferroptosis, RAP1, MAPK, HIF1, TNF, focal adhesion, and prostaglandin pathways showed as a significant molecular mechanism (Figure 3 and Table 1).

**TABLE 1:** The top 10 genes most express differentiation in glioblastoma cancer stem cells.

Genes	*p*‐value	LogFC
Up‐regulated genes		
*MSMP*	1.96E‐06	23.97836
*SLC24A3*	7.22E‐09	23.11268
*SOX2*	2.24E‐10	17.31233
*FGD4*	2.13E‐11	13.75152
*KCNIP3*	9.71E‐09	11.6337
*CNTNAP3*	1.08E‐07	11.05958
*CNTNAP3B*	1.54E‐08	10.54311
*PRICKLE1*	1.27E‐12	10.54134
*PLA2R1*	2.45E‐10	10.26455
*XLOC‐004423*	2.09E‐10	10.26177
Down‐regulated genes		
*ARHGEF7*	4.47E‐02	−0.07322
*FADD*	4.41E‐02	−0.07419
*MTRNR2L6*	3.76E‐02	−0.0744
*PRNP*	4.92E‐02	−0.07525
*FLJ43681*	3.98E‐02	−0.07557
*RN28S1*	4.94E‐02	−0.07559
*C1orf85*	3.57E‐02	−0.07725
*ARIH2*	4.87E‐02	−0.0787
*TMEM222*	4.52E‐02	−0.07872
*PRPF4*	3.50E‐02	−0.07879

### 3.2 GO analysis

**FIGURE 4:**
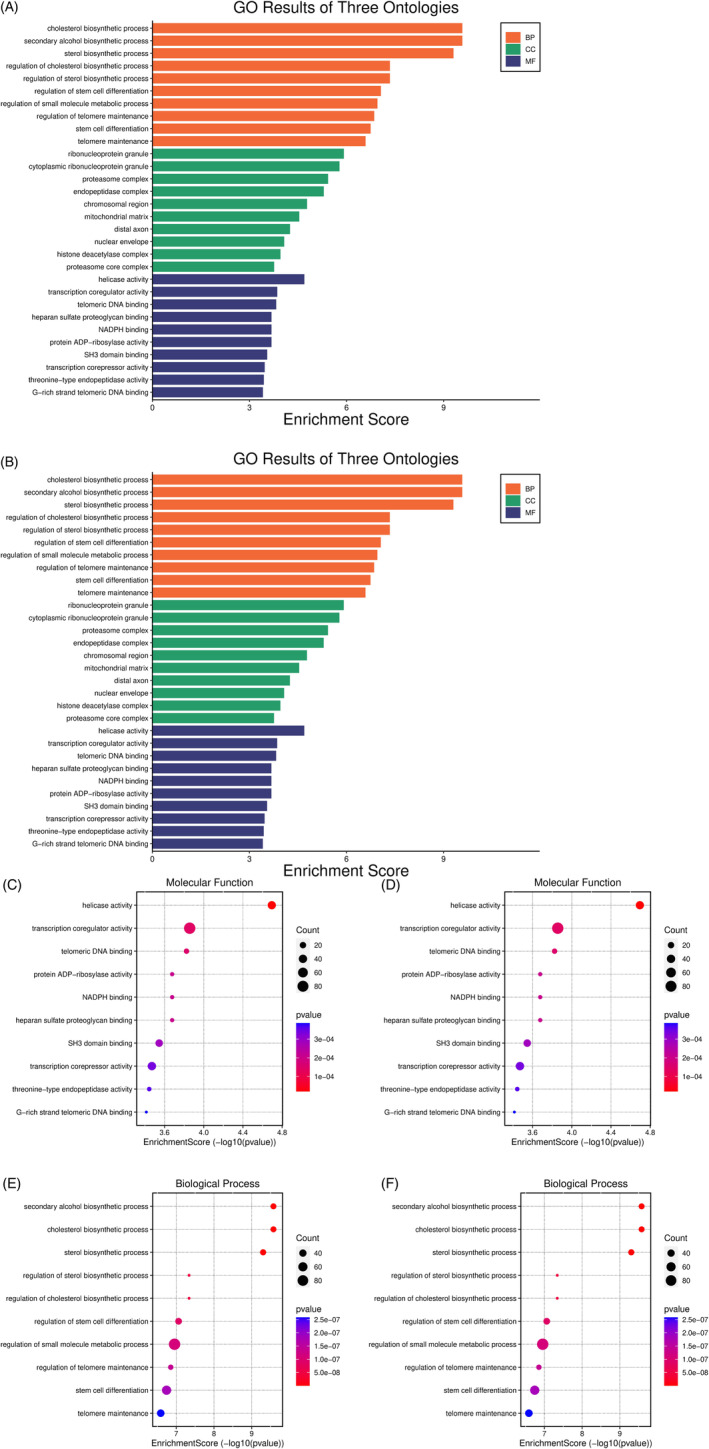
The communication network between biological processes in glioblastoma cancer stem cells with high‐ and low‐expressions was plotted using the shiny gene ontology database. Accordingly, in this network, the size of the circles and the color intensity between them show more significance. (A): upregulated genes; (B) downregulated genes. (C and E) Upregulated biological processes and molecular functions. (D and F) downregulated biological processes and molecular functions.

In the BPs of cell cycle pathways, processing of cellular processes, positive self‐regulation in microenvironments pathways, regulation of biosynthetic processes and intracellular transitions, MFs of phosphates, freezled junctions, integrin junctions, endothelial vascular growth factor, and cytokine activity was present. The low‐expression genes regulate phosphorus metabolism, regulate cell motility, and respond to cellular stress and organic molecules. Organization of cellular organs and modification of macromolecule structures were involved in BPs. NADH dehydrogenase and adenosine triphosphate (ATP) activities were involved in MFs (Figure 4).

### 3.3 Protein network analysis

**FIGURE 5:**
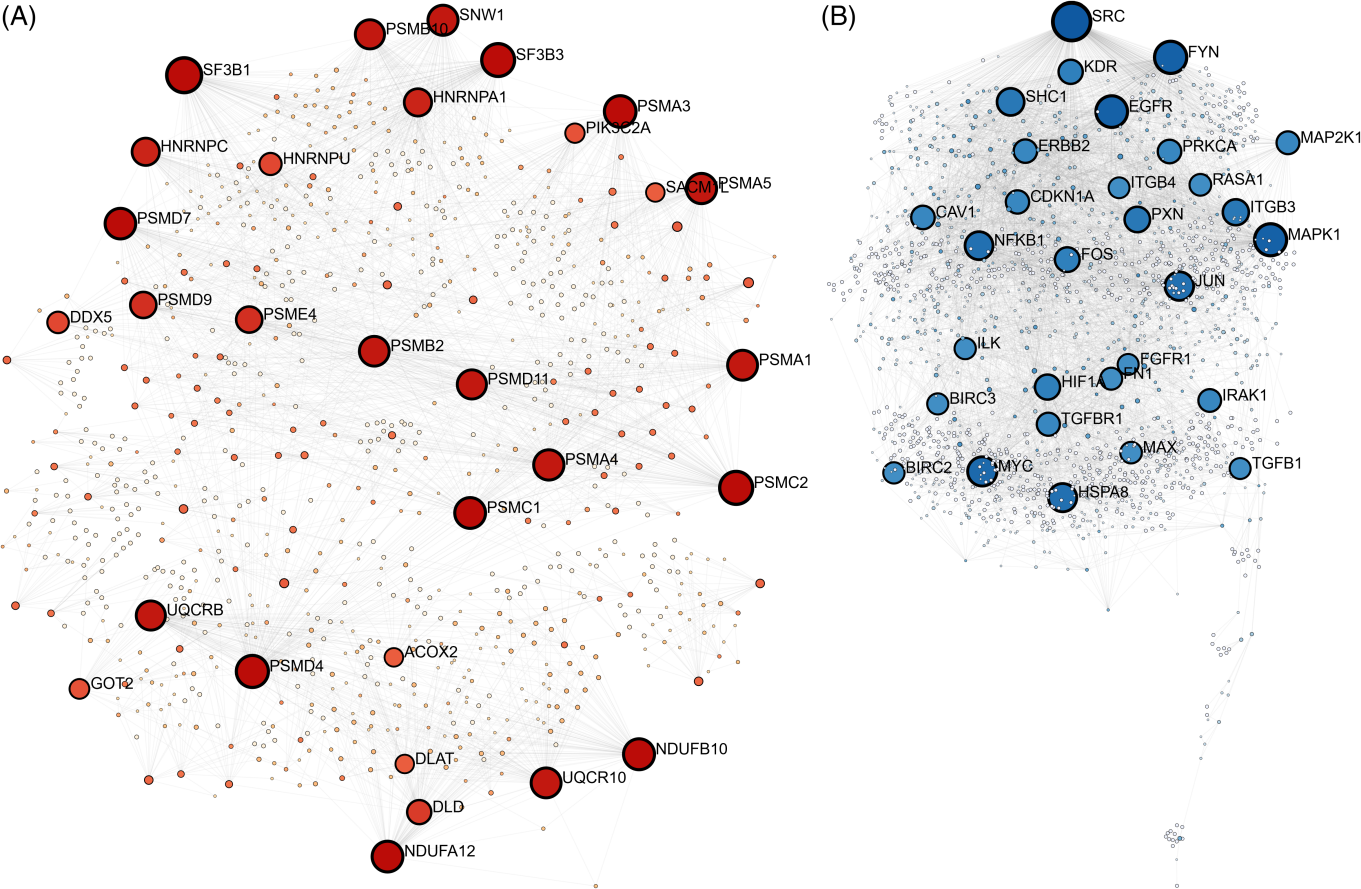
Protein network between high‐ (A) and low‐expression (B) genes. Phosphatidylinositol pathways, cholesterol biosynthesis, carbon cycle metabolism in cancer, and glycolysis have been identified.

We examined the genes with high‐ and low‐expression at this stage that were involved in critical microenvironment pathways. Accordingly, 129 nodes and 236 edges are formed in the protein network with increased expression, and 67 nodes and 175 edges in the protein network with low expression (Figure 5).

### 3.4 Evaluation of candidate genes in clinical data related to GBM CSCs

**FIGURE 6:**
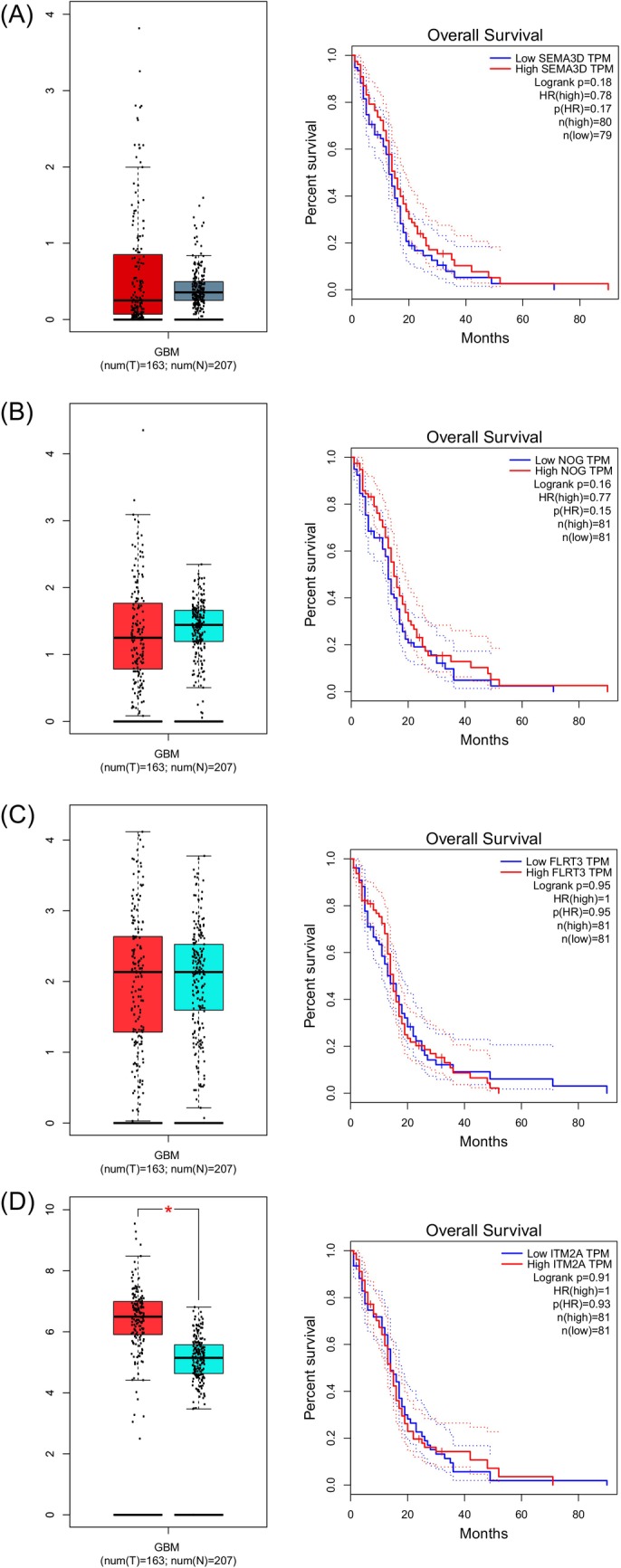
Expression and survival curves in candidate genes with high and low expression are identified, related to *SEMA3D* (A), *NOG* (B), *FLRT3* (C), and *ITM2A* (D) genes.

In this section, genes and protein products related to the activity of GBM CSCs were evaluated. Accordingly, *integral membrane protein 2A* (*ITM2A*), *fibronectin leucine‐rich transmembrane protein 3* (*FLRT3*), noggin (*NOG*), and *SEMA3D* genes increased expression. Interestingly, all of these genes throughout approximately the same period (about 20 months), showed a significant reduction in the survival analysis (Figure 6).

## 4 DISCUSSION

Neurosurgeons and doctors have been quite worried about GBM. Recurrence happens despite extensive chemotherapy and high‐dose radiation therapy, which lead to the removal of tumor tissue by a complex surgical procedure.^12^ Among the most common reasons for recurrence, cancer cells associated with GBM tumor tissue often persist in the patient's body after surgery. Radiation and chemotherapy can cause these cells to develop resistance.^13^ The ability of a small number of GBM CSCs to differentiate into massive tumors and for all of these tumor cells to originate from GBM CSCs is a direct result of stem cells' two primary properties, self‐regeneration and differentiation.^14^ It is necessary to change the type of radiation therapy or the combination of chemotherapy medications, as the patient's previous treatments were ineffective.^14^, ^15^ It is crucial to identify biomarkers linked to GBM CSCs because no one can have GBM and also risk other cancers.^12^, ^13^, ^14^, ^16^, ^17^, ^18^ Although the precise molecular pathways connecting GBM CSCs and tumor‐associated macrophages (TAMs) are unknown, their interaction promotes GBM growth.^13^, ^14^, ^15^, ^16^, ^17^, ^18^, ^19^, ^20^ In this study, Yin et al.^21^ shown that the zinc finger protein arsenite‐resistant protein‐2 (ARS2) is crucial for the early development of mammals and has an important function in preserving GBM CSCs and M2‐like TAM polarity. In order to control the self‐regeneration and tumorigenesis of GBM CSCs, ARS2 activates MGLL, a new transcription target gene that encodes monoacylglycerol lipase (MAGL). This process involves the production of prostaglandin E2 (PGE2), the activation of β‐catenin in CSCs, and the polarity of TAM, which in turn stimulates pseudo‐M2.^21^ The M2‐like signature genes were also discovered by Yin et al.^21^ These genes were responsible for the MAGL‐specific inhibitor JZL184's ability to prevent PGE2 synthesis, which in turn boosted survival in a mouse xenograft transplant model. Overall, Yin et al.^21^ found that inhibiting ARS2/MAGL signaling offers a potential new therapy approach for GBM patients by limiting the link between GBM CSCs and TAMs. Brain cancer is just one of many disorders for which multivariate evaluations of worldwide expression profiles provide reliable prognoses. Using patients' clinical information and expression data, Takashima et al.^22^ conducted multivariate analyses on the status of glioma stem cells (GSCs), molecular target therapy (MTT), potential glioma biomarkers (PGBs), and epithelial–mesenchymal transition (EMT) to identify candidates for GBM prognostic markers. Data from the Cox test and random proportional hazard regression analysis demonstrated that DSG3, CLDN1, CDH11, FN1, histone deacetylase 3 (HDAC3)/PTEN, L1CAM, OLIG2, TIMP4, IGFBP2, and GFAP were significantly variable.^22^ Prognostic prediction methods that may differentiate between GBM survival curves were also a part of the investigations.^22^ An intricate web of genetic communication networks also includes the genes mentioned above as well as HDAC1, FLT1, EGFR, MGMT, PGF, STAT3, SIRT1, and GADD45A.^22^ Good prognosis was associated with MESmiddle, GSClow, and PGBlow, whereas MEShigh GLIlow, MEShigh MTTlow, and PGBhigh all showed poor prognosis according to survival curve analysis.^22^ According to these findings, evaluating EMT and GSC might help predict the prognosis of GBM, which in turn can lead to the creation of new treatments and potential markers for the prognosis of GBM.^19^, ^20^, ^21^, ^22^ Because of their extremely aggressive character and resistance to traditional therapy, a subgroup of cancer cells known as mesenchymal GBM CSCs is produced.^19^ Recent research has pointed to aldehyde dehydrogenase 1A3 (ALDH1A3) as a key factor in preserving mesenchymal GBM CSC properties,^23^ but the overall processes of ALDH1A3 ectopic expression are still a mystery. In mesenchymal GBM CSCs, the ubiquitin‐9‐specific protease (USP9X) was discovered as a deubiquitinase in collaboration with ALDH1A3 by Chen et al.^23^ ALDH1A3 and polyubicoetilate interacted with USP9X, stabilizing it. Furthermore, it was demonstrated that USP9Xhi cells that were categorized by fluorescence activated cell sorter were more likely to include GBM CSCs that exhibited significant ALDH1A3 activity and tumorigenic solid capability.^23^ Loss of autoimmune capacity and tumorigenesis of GBM CSCs, which may be strongly linked to ALDH1A3 expression, were caused by decreased USP9X, which in turn lowered ALDH1A3^23^ Furthermore, it was found that the USP9X WP1130 inhibitor had a substantial impact on ALDH1A3 degradation and demonstrated promising therapeutic results in models of orthopedic xenograft transplantation produced from GBM CSCs.^23^ The mesenchymal subtype of GBM patients were significantly impacted by USP9X, and there was a strong correlation between USP9X and ALDH1A3 expression in human GBM prototypes.^23^ Therefore, these results point to USP9X as a possible target for GBM CSCs‐based treatment and a critical deubiquitinase for ALDH1A3 protein stability.^19^ Microenvironment pathways, which provide the energy for GBM CSCs activity, have received insufficient attention despite these investigations. Using bioinformatics as a lens, we identified key biomarkers associated with secretory GBM CSCs and conducted our investigation accordingly. We observed that whereas the MINPP1, SQLE, and DHCR7 genes are highly expressed, the ENO2 and HK1 genes are moderately expressed. In the cholesterol biosynthesis route, DHCR7 has the potential to decrease the double bonding of two intermediates: C7‐C8‐cholesta‐5,7‐diane‐3‐beta‐ol (7‐dehydrocholesterol/7‐DHC) and cholesta‐5,7,24‐trine‐3‐beta‐ol.^24^, ^25^ While research on this gene has focused on other malignancies, it has not been explicitly found in GBM CSCs. There is evidence linking vitamin D insufficiency to an increased risk of cancer.^25^ The enzymes DHCR7 and CYP2R1 play critical roles in the metabolism of vitamin D.^25^ Vitamin D may have a role in cancer risk due to its relationship with DHCR7 and CYP2R1 gene polymorphisms, which is why scientists are looking into this. However, there is a lack of consistency in the findings. The link between cancer risk and the DHCR7 and CYP2R1 SNPs was investigated in a comprehensive investigation by Wen et al.^25^ Using odds ratios, they determined the risk of cancer associated with each SNP. In the meta‐analysis of five SNPs (DHCR7 rs12785878, rs1790349 SNP, CYP2R1 rs1074116579, SYP2R1 rs1074116579, and rs1074126574), 12 designed case–control studies with 23 780 cases and 27 307 controls were ultimately identified.^21^ There was a statistically significant association between cancer risk and the DHCR7 rs12785878 SNP across the board in the study population.^25^ One study found that the DHCR7 rs1790349 SNP increased cancer risk in white people.^25^ Furthermore, the CYP2R1 SNPs rs12785878, rs1790349, and rs12794714 may serve as biomarkers for cancer susceptibility, and the rs12794714‐A allele was linked to a decreased risk of colon cancer.^25^

Enzymes unique to phosphatidylcholine activate diacylglycerol and inositol 1,4,5‐triphosphate, 2 s messenger chemicals necessary for trophoblast and placenta formation. Laser spectroscopy has seen tremendous growth in usage over the past several decades, finding theoretical and experimental uses in fields including biomolecular research and medical treatment. The properties of random lasers were tested in two groups of human breast xenograft transplant tissues^26^, ^27^ to assess the viability of a random instrument to mark the effect of PLCD1 gene therapy on breast cancer. The AdHu5‐EGFP and AdHu5‐PLCD1 groups showed coherent and incoherent random laser regimes, respectively.^27^ The random coherent laser propagation that results from the increased light scattering caused by the intrinsic disruption of breast tumor tissues^27^ is a common occurrence in tumor specimens. Tumor tissues treated with PLCD1 showed a more orderly spatial organization of breast tumor cells, as validated by hematoxylin and eosin staining.^27^ A protein whose RhoGAP activities and scaffolding contribute to tumor suppressor roles are encoded by the DLC1 gene, which is controlled in many forms of cancer by both genetic and nongenetic pathways.^28^ The DLC1 START domain's function, also known as STAR‐associated fat transfer or DLC1‐START, is unclear beyond its binding to Caveolin‐1.^28^ The potential lipid ligand for DLC1‐START is unknown;^28^ however, other START domains have a key function that requires binding lipids. In addition to Caveolin‐1, Sanchez‐Solana et al.^28^ demonstrated that DLC1‐START binds to the PLCD1 protein, and they also identified PS as a lipid ligand for DLC1‐START.The connection between DLC1 and Caveolin‐1 and PLCD1 is facilitated in part by PS binding.^28^ Seven cancer‐associated mutations in DLC1‐START were found to decrease tumor suppressive activity, highlighting the relevance of these actions for carcinogenesis.^28^ When comparing PLCD1 expression in normal esophageal epithelial cells to esophageal squamous cell carcinoma (ESCC) cells, Hörer et al.^25^, ^26^, ^27^, ^28^, ^29^ discovered a significant decrease. Furthermore, TE‐1 and EC18 cells' abilities to invade, migrate, and proliferate were diminished when PLCD1 was positively regulated.^25^ A negative correlation between PLCD1 activity and the expression of catenin, C‐myc, cyclin D1, MMP9, and MMP7 was discovered.^25^ At long last, in vivo evidence that PLCD1 activation inhibits ESCC proliferation has been found.^29^

MINPP1 controls the amounts of inositol pentose phosphate and inositol hexachord phosphate within cells by acting as a 5‐phosphoinositide and 5‐phosphoinositide 6‐phosphatase.^30^ It dephosphorylates 2,3‐bisphosphoglycerate (2,3‐BPG) to create phospho‐D‐glycerate without forming 3‐phosphoglycerate, and it also functions as a 2,3‐bisphosphoglycerate 3‐phosphatase.^30^ An crucial biological function in hepatocellular carcinoma (HCC) was discovered by Chen et al.^31^ via the glycolytic bypass microenvironments pathway and a gene named MINPP1. In addition, MINPP1 is controlled in HCC and may inhibit the migration and proliferation of tumor cells.^31^ In HCC, miRNA‐30b‐5p stimulates tumor cell proliferation by a glycolytic bypass.^31^ Even more crucially, miRNA‐30b‐5p has the potential to drastically lower MINPP1 expression.^31^ The miRNA‐30b‐5p/MINPP1 axis was found to speed up the glucose‐to‐lactate and 2,3‐BPG conversion in microenvironment testing. The ability of miRNA‐30b‐5p and MINPP1 to induce carcinogenesis by regulating glycolytic bypass is not present in HCC cells.^31^ But miRNA‐30b‐5p and MINPP1 greatly expanded, tumor cell migration enhanced, and glycolytic bypass encouraged, once the hepatitis B virus (HBV) entered these cells. It was shown that miRNA‐30b‐5p expression is increased during HBV infection due to the interaction between the HBV P protein and FOXO3.^31^ Optimal survival of hepatitis C virus‐positive patients with HCC was associated with increased MINPP1 expression, leading to slower disease development, according to bioinformatics analysis of a large cohort dataset.^31^

Some examples of hexoses that can be phosphorylated to hexose 6‐phosphate are D‐glucose 6‐phosphate and D‐glucosamine 6‐phosphate, which can be produced by the HK1 enzyme.^32^ Furthermore, it functions as a receptor for bacterial peptidoglycan patterns, which it uses to contribute to inflammation and innate immunity.^32^ Bacterial peptidoglycan N‐acetyl‐D‐glucosamine inhibits HK1 action when released into the cytosol, leading to its detachment from the outer mitochondrial membrane and activation of NLRP3 inflammation.^33^, ^34^ The researchers Zhang et al.^31^ shown that in DLD1 and HCT8, HK1‐cDNA was able to express HK1 and si‐HK1 was able to block HK1 expression. Colorectal cancer cells exhibited enhanced migration and invasion upon ectopic HK1 expression, whereas rat models showed higher lung metastasis.^35^ Further, colorectal cancer cells migrated and invaded by HK1‐induced Snail2 expression, which boosted epithelial–mesenchymal transmission and cancer progression.^35^ An aberrant energy metabolism characterized by an over‐reliance on aerobic glycolysis, often known as the Warburg effect, is common in tumors [3236]. In order to develop more effective cancer treatments, it is crucial to understand these energy shifts in malignant tumors. The impact of the initial and rate‐limiting inhibition of the glycolytic HK isoenzymes HK1 and HK2 on tumor growth was studied by Tseng et al.^36^ Expressed HK1 and HK2 in inverse association in human cancer cells was reported earlier.^36^ In fact, quenching HK1 impaired aerobic respiration and increased glycolysis without influencing ATP production,^36^ but it did cause an epithelial–mesenchymal phenotypic change in cervical cancer cells, which sped up tumor growth and metastasis in both in vitro and in vivo tests. A decrease in citrate synthase levels and an increase in HK2 and lactate dehydrogenase‐1 expression were linked to these alterations in the microenvironment.^32^ In instance, abnormal energy metabolism was virtually directly associated with HK2 overexpression when HK1 failed.^36^ Also, 2‐deoxyglucose (2‐DG) inhibited cell proliferation whereas HK1‐quenched cells exhibited robust glucose‐dependent growth.^36^ These findings suggest that reducing HK1 expression, but not HK2, changes energy metabolism and causes an EMT phenotype, which in turn enhances tumor malignancy and makes cancer cells more sensitive to 2‐DG inhibition.^36^ Furthermore, it implies that malignancies with strong glycolytic activity should be the only ones treated with glycolytic inhibitors.^37^ Clinical characteristics of patients with ovarian cancer were associated with elevated HK1 expression, according to research by Li et al.^37^ Analysis of survival curves revealed that ovarian cancer patients overexpressing HK1 had a significantly worse chance of surviving.^37^ Furthermore, HK1 may serve as a standalone biomarker for the dismal outcome experienced by ovarian cancer patients, as suggested by both univariate and multivariate analyses.^37^ Through MAPK/ERK signaling, HK1 inhibits glucose absorption, lactic acid generation, ATP production, invasion, and migration.^37^

On several neurons in the central nervous system (CNS), ENO2 has neurotrophic and neuroprotective effects.^37^ Increased cell survival is a result of its calcium‐dependent binding to cultured neocortical neurons.^37^ Heart attacks, brain tumors, brain trauma, and Creutzfeldt–Jakob disease are associated with significantly elevated ENO2 levels.^37^ Research has demonstrated that ENO2 is controlled in a variety of tumor types, such as sarcoma, pancreatic cancer, leukemia, melanoma, renal cancer, esophageal cancer, and cervical cancer.^37^, ^38^ Overexpression of ENO2 was also associated with a worse prognosis for lung cancer patients. Consequently, ENO2's prognostic power was significantly higher in lung adenocarcinoma patients.^39^ When it comes to glycolysis in the microenvironment, ENO2 is an essential enzyme. Researchers Zheng et al. found that pancreatic tissues had a marked overexpression of ENO2, and that this overexpression was linked to both poor prognosis and metastasis in pancreatic cancer patients.^36^ Enzymatic activity, cellular metabolism, and progression are regulated by K394, a significant acetylation site in ENO2.^40^ Furthermore, by eliminating ENO2, the spread of tumors and metastases to the liver could be halted.^36^ Expression of wild‐type ENO2 was found to decrease tumor malignancy, in contrast to mutant K394 mimetic acetylation.^40^ For the most part, HDAC3 was an ENO2 deacetylase, while for CBP, it was an acetyltransferase.^40^ Furthermore, they demonstrated that deacetylation mediated by HDAC3 causes ENO2 activation and enhanced glycolysis.^36^ In a dose‐ and time‐dependent manner, insulin‐like growth factor‐1 reduces K394 acetylation, activates ENO2, and accelerates the PI3K/AKT/mTOR pathway of HDAC3 phosphorylation in S424.^36^ Furthermore, acetylation modulates glycolysis, which in turn negatively influences ENO2 activity in pancreatic cancer metastasis, as recently shown by Zheng et al.^40^ Therefore, a potential method to prevent pancreatic cancer could be to stop the insulin‐like growth factor‐1‐induced ENO2 deacetylation.

In the current study, via the GEPIA database (dependent on the the Cancer Genome Atlas [TCGA] clinical database), we found that *ITM2A*, *FLRT3*, *NOG*, and *SEMA3D* expressions significantly increased. All genes are relatively directly related to the mortality rate of patients with GBM and reach less than 10% in about 20 months, which calls for their high importance for laboratory and clinical evaluations. These genes are still unclear in GBM CSCs and need further clarification.

## AUTHOR CONTRIBUTIONS

**Ehsan Jangholi:** Conceptualization; methodology; formal analysis; investigation; validation; writing – original draft; writing – review and editing. **Hoda Ahmari Tehran:** Methodology; writing – review and editing; writing – original draft; validation; data curation. **Afsaneh Ghasemi:** Methodology; data curation; validation; writing – review and editing; writing – original draft. **Mohammad Hoseinian:** Writing – review and editing; writing – original draft; validation; data curation; methodology. **Sina Firoozi:** Writing – original draft; writing – review and editing; validation; data curation. **Seyed Mohammad Ghodsi:** Writing – review and editing; writing – original draft; methodology; conceptualization; validation; investigation. **Mona Tamaddon:** Writing – review and editing; writing – original draft; methodology; validation. **Ahmad Bereimipour:** Writing – review and editing; writing – original draft; validation; methodology; investigation; formal analysis. **Mahmoudreza Hadjighassem:** Writing – review and editing; writing – original draft; validation; methodology; conceptualization; supervision.

## FUNDING INFORMATION

This research was supported by grants from Brain and Spinal Cord Injury Research Center, Neuroscience Institute, Tehran University of Medical Sciences (grant number: 57665).

## CONFLICT OF INTEREST STATEMENT

The authors have stated explicitly that there are no conflicts of interest in connection with this article.

## ETHICS STATEMENT

This study was approved by the Research Ethics Committees of the Neuroscience Institute, Brain and Spinal Cord Injury Research Center (approval code: IR.TUMS.NI.REC.1401.023).

## Data Availability

The analysis code and datasets used here are available from the corresponding author on request.
